# Editorial: Pruritus Medicine

**DOI:** 10.3389/fmed.2021.763667

**Published:** 2021-09-28

**Authors:** Sonja Ständer, Yan-Gang Sun, Martin Schmelz

**Affiliations:** ^1^Department of Dermatology, Center for Chronic Pruritus, University Hospital Münster, Münster, Germany; ^2^State Key Laboratory of Neuroscience, Center for Excellence in Brain Science & Intelligence Technology, Institute of Neuroscience, Chinese Academy of Sciences, Beijing, China; ^3^Department Experimental Pain Research, Medical Faculty Mannheim, University of Heidelberg, Mannheim, Germany

**Keywords:** pruritus, itch, prurigo, neuronal sensitization, atopic dermatitis

Pruritus is a burdensome symptom and defined as chronic when lasting for at least 6 weeks according to the International Forum for the Study of Itch (IFSI) (1). Chronic pruritus (CP) is an intrinsic symptom of multiple diseases such as inflammatory, infectious and autoimmune dermatoses, hepatobiliary and chronic kidney diseases, systemic and cutaneous lymphoma, solid neoplasms, neurological and mental conditions. As a disease symptom, CP is thus highly prevalent in the general population Weisshaar. However, the neurobiology of CP is complex and its causal link to the underlying diseases remains unclear as described in two papers on encoding Schmelz and central processing of pruritus Najafi et al. Experimentally, pruritus can be induced by electrical stimulation Solinski and Rukwied, Meijer et al. and reduced by affective touch. Clinically, cholestatic pruritus might be related to bile salts, steroid metabolites, serotonin, protease-activated receptor agonists or most likely lysophosphatidic acids Langedijk et al., but probably not by endogenous opioids as shown by Düll et al. The long list of potential mediators reflects the current dilemma in pruritus research: many potential mediators have been identified but unfortunately, the clinical validation in representative patient cohorts is pending.

Still, animal studies are indispensable to identify novel mechanisms. Donglang et al. guide us through translational aspects of these animal models. Liu et al. describe an acute itch model by intradermal injection of low-dose formalin which may be employed as a screening tool for potential anti-itch drugs. Also, novel topics such as the role of the gut microbiota in pruritus was investigated in an animal model and described here (Li Y. et al.). Jia et al. even describe a novel functional factor which is relevant for itch signal processing, and show that the transcription factor ZBTB20 (Zinc Finger And BTB Domain Containing 20), which is expressed in small primary sensory neurons, could modulate itch regulating TRP channels.

Itch induction in the skin is frequently based on inflammation and neuroimmune mechanisms. The latter refers to the bidirectional communication between cells releasing inflammatory mediators such as interleukin (IL) 4 or IL 31, neurotrophins such as nerve growth factor and neuropeptides released by sensory nerve fibers. The implications of such neuroimmune interactions is presented in three papers by Ruppenstein et al., Kabashima and Irie, and Nemmer et al. Atopic dermatitis is the most frequent inflammatory dermatosis; Legat gives an overview on its pathophysiology including the relevant neuroimmune mechanisms and also provides information on the corresponding current treatment options. While atopic dermatitis is already well-understood regarding its pathophysiology, other pruritic dermatoses, systemic diseases and neuropathic entities are not. However, in past years, several entities reached a broader understanding and novel mechanisms or therapies have been identified. Accordingly, our authors report on the latest knowledge in chronic prurigo Zeidler et al., scabies Ständer and Ständer, pruritus related to therapy with immune checkpoint inhibitors Salinas et al., chronic kidney disease Krajewski et al. and neuropathic diseases Pereira et al. as well as sensitive skin condition (Brenaut et al.), and vulvar pruritus Raef and Elmariah. For poorly understood cases of pruritus cannot be linked to inflammatory or neuropathic mechanisms Misery extends the concept of nociplastic changes in the pain field and suggests using the term pruriplastic pruritus for them.

Mental factors are important for itch perception and its progression. Lüßmann et al. describe that itch intensity and certain facets of mindfulness were associated with itch catastrophizing in AD patients. On the other hand, distraction might help to reduce itch as investigated by van Laarhoven et al. by training volunteers to reduce attention toward itch. Pruritus is a subjective symptom without objective measurement. Accordingly, patient reported outcomes are still the gold standard in the assessment of the course. However, the complex process of international harmonization and validation is still ongoing. Stepień and Reich contribute to this topic by framing severity levels in a novel questionnaire.

In sum, this collection of articles combines several up to date aspects of the neurobiology and clinics of pruritus, including also new methods and innovative concepts in itch research.

## Author Contributions

All authors listed have made a substantial, direct and intellectual contribution to the work, and approved it for publication.

## Funding

This article was supported by the German Research Foundation (DFG/NO FOR2690 to SS and MS).

## Conflict of Interest

The authors declare that the research was conducted in the absence of any commercial or financial relationships that could be construed as a potential conflict of interest.

## Publisher's Note

All claims expressed in this article are solely those of the authors and do not necessarily represent those of their affiliated organizations, or those of the publisher, the editors and the reviewers. Any product that may be evaluated in this article, or claim that may be made by its manufacturer, is not guaranteed or endorsed by the publisher.
